# A Strategy to Optimize the Performance of Bio-Derived Carbon Aerogels by a Structuring Additive

**DOI:** 10.3390/nano10091811

**Published:** 2020-09-11

**Authors:** Marcelina Kubicka, Monika Bakierska, Krystian Chudzik, Marcin Molenda

**Affiliations:** Faculty of Chemistry, Jagiellonian University, Gronostajowa 2, 30-387 Krakow, Poland; lis@chemia.uj.edu.pl (M.K.); krystian.chudzik@doctoral.uj.edu.pl (K.C.)

**Keywords:** Li-ion battery, anode material, carbon aerogel, renewable resource, starch, structuring additive, gum arabic

## Abstract

In this work, we investigated the influence of gum arabic (GA) as a structuring additive, on the electrochemical behavior of bio-derived carbon aerogels (CAGs). Modified carbonaceous materials were prepared by the gelatinization process of potato starch (PS) with the addition of GA in various quantities, followed by the thermal treatment of the obtained gels in an inert gas atmosphere. The obtained anode materials were examined by X-ray diffraction (XRD), elemental analysis (EA), galvanostatic charge/discharge tests (GCDT), extensive cycling (LT-GCDT) and cyclic voltammetry (CV) methods. The highest electrochemical performance was achieved for carbon aerogel material, in which 1% *w*/*w* GA was added. The results showed that the proper composition of carbon precursor with a structuring promoter improves the rheological properties of starch gel and stabilizes the final aerogel structure affecting CAG functional properties.

## 1. Introduction

Nowadays, the main share of the Li-ion market is dominated by cells based on carbon anodes. This phenomenon results mostly from a relatively low price and a reasonable capacity of carbonaceous materials (372 mAh/g for graphite [1]). Despite the constant search for a better anode, graphite (natural or synthetic) is still the most relevant in Li-ion battery [2]. However, this material has also some disadvantages. Among the flaws of natural graphite are its limited resources and non-renewability. Furthermore, the process of extracting graphite from the earth can be hazardous and impoverish the natural surroundings. On the other hand, artificial graphite requires harsh synthesis conditions and big energy expenditure which leads to the greater emission of harmful gases [3,4]. Thus, along with the quest for low-pollution variants in the world facing a climate catastrophe, Li-ion industry, as a critical part of the energy production system, has to follow up to the upcoming changes and impose the environmental requirements [5,6]. Hence, there is a need to find and develop other alternatives for conventional anode material, that allow to harness the renewable energy [7].

There are several renewable resources that can be converted into carbonaceous materials such as rice grains [8], pine cones/needles, oak leaves, orange peels [9], pollens [10], mushrooms [11] or algae [12]. Nonetheless, these applied in Li-ion cells as negative electrode can also often require a high pressure synthesis condition [7]; complex, multi-step treatment [11]; or very high temperatures during thermal treatment [11,13]. Therefore, among them, the interesting direction in Li-ion batteries studies is the application of starch-based carbon aerogels (CAGs), and what is associated with their unique features that make them competitive for energy storage systems in terms of their properties, economy and ecology [14,15,16,17,18]. They are obtained through eco-friendly, water-based synthesis. Their facile preparation process including gelatinization, solvent exchange, low-temperature drying under ambient pressure and pyrolysis, requires relatively low energy expense (carbonization at maximum 700 °C), in comparison to the currently used carbonaceous anodes (thermal treatment at 2500 °C for synthetic graphite [4] and over 1000 °C for many types of hard carbons [19]).

Another viable natural source for Li-ion batteries can be the gum-like substance, oozing from branches and stems of Acacia Senegal mature trees—gum arabic or gum Acacia (GA) [20]. Its chemical composition varies depending on the source, nonetheless, it mainly consists of a polysaccharide mixture including galactose, arabinose, rhamnose and 4-O-methyl glucuronic acid. Although, most often, gum arabic can be found in the confectionary industry [20], it can be also incorporated in the energy storage systems including its use as a binder for electrode composites [21], a precursor for pyrolytic carbon layers in advanced oxide anodes [22] or a self-healing agent for Si-based novel anodes [23].

Our recent studies have led us to an alternative application of gum arabic as a structuring additive for bio-derived, starch-based carbon aerogels for Li-ion batteries, which is the subject of the present work. However, the systems consisting of starch and non-starch hydrocolloid gums has been widely studied, since they are extensively used in food application [24,25,26,27,28,29,30], they have never been investigated in terms of their usage in energy storage systems. The addition of gum to starch is one of the most popular ways to modify the starch properties (including maintaining the desirable structural, textural and rheological characteristics) and the interactions between these two components are quite well understood. Gum arabic is known for having functionally structural components and that is why it has been reported as an encapsulating and emulsifying agent that plays an important role in the structuring and thickening of the starch-based system while modifying its rheological properties [25,27,30]. Therefore, the GA additive during carbon aerogel synthesis should influence the properties of the resulting organic starch aerogels and hence the carbon materials derived from such a precursor, which ought to clearly translate into electrochemical results. Undoubtedly, the enhanced electrochemical properties of modified CAG materials can open another interesting usage for this versatile, natural resource. An evaluation of the electrochemical performance of GA-structured carbon aerogels as a novel, eco-friendly Li-ion anodes is the main concern of presented study.

## 2. Materials and Methods

The modified carbon aerogels, based on potato starch (Sigma Aldrich, Saint Louis, MO, USA), were obtained via a gelatinization process described in detail in our previous work [14,15,16,17,18]. A gum arabic (Avantor Performance Materials Poland S.A., Gliwice, Poland, 99%), as a structuring additive, was introduced during starch hydrogel preparation, in quantities of 1% *w*/*w*, 5% *w*/*w*, 10% *w*/*w* and 20% *w*/*w*. Briefly, potato starch with gum arabic were dispersed in distilled water and the suspensions were stirred and heated in an oil bath until the gelatinization temperature was reached. Then, the samples were poured over with ethanol solution (Avantor Performance Materials Poland S.A., Gliwice, Poland, 96%) and left for 5 days in order to replace the water trapped inside the pores. After the solvent exchange, the obtained gels were dried at 50 °C under atmospheric pressure and then subjected to a thermal treatment process at 700 °C, under argon (Air Products, Allentown, PA, USA, 99.999%) flow, for 6 h, with a constant heating rate (2 °C/min).

The phase purity of the studied materials was investigated by X-ray powder diffraction (XRD) using a Bruker D2 PHASER diffractometer (Billerica, MA, USA) with a Cu lamp K_α1_ radiation, λ = 0.154184 nm. By the application of the elemental analysis (EA)—performed on the micro analyzer Vario MICRO cube coupled with microbalance (Elementar)—the carbon/hydrogen/nitrogen (CHN) content was measured.

The galvanostatic charging/discharging cycling tests (GCDT) were conducted on the ATLAS 1361 MPG&T multichannel testing system (ATLAS–SOLLICH, Rębiechowo, Poland), to investigate the impact of GA addition on the electrochemical performance and stability of CAG-based cells. For the most effective sample, the additional cycling at 3 °C and 61 °C were performed. All measurements were carried out between 0.001 and 3.0 V, under different current loads (from C/20 to 20C), with metallic lithium (Sigma Aldrich, Saint Louis, MO, USA, 99.9%) as a reference, the solution of 1M lithium hexafluorophosphate (LiPF_6_) dissolved in a mixture of ethylene carbonate with diethyl carbonate (50/50 v/v, Sigma-Aldrich, Saint Louis, MO, USA, battery grade) as an electrolyte, and a trilaminate of polypropylene/polyethylene/polypropylene film (Celgard 2325) (Celgard LLC, Charlotte, NC, USA) with two porous glass microfiber filters (Whatman GF/F) (Sigma Aldrich, Saint Louis, MO, USA) as separators. The electrodes were fabricated by mixing 90 wt% of active material with 10 wt% of binder-polyvinylidene fluoride (Sigma Aldrich, Saint Louis, MO, USA). The mixture was prepared as a slurry in N-methyl-2-pyrrolidone (NMP) solvent (Sigma Aldrich, Saint Louis, MO, USA, ≤99.5%), spread onto copper foil and dried in an air-oven at 120 °C for 48 h under ambient pressure. The R2032 coin-type half cells were assembled in an argon-filled glove box (MBraun glove box, Garching, Germany) with a high-purity atmosphere (H_2_O and O_2_ < 0.1 ppm). For all cells, the mass loading of active material on the electrode was equal to 1.18 ± 0.09 mg/cm^2^.

Additionally, the cyclic voltammetry (CV) measurements were carried out on the AUTOLAB PGSTAT302N/FRA2 potentiostat/galvanostat (Metrohm Autolab, Utrecht, The Netherlands), starting from an open circuit voltage (OCV), by applying 0.1 mV/s scan rate, in the voltage range from 0.001 V to 3 V.

## 3. Results and Discussion

**Figure 1:**
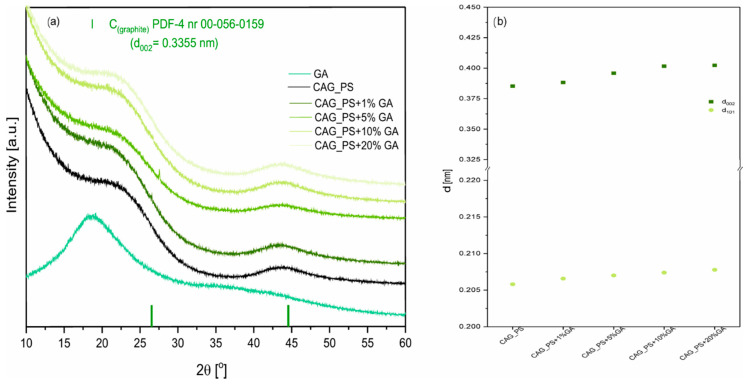
(**a**) X-ray powder diffraction patterns of modified carbon aerogel materials (CAG_PS) with gum arabic (GA) and (**b**) the interplanar spacing values for the obtained samples.

Figure 1a presents the X-ray diffraction patterns of modified carbon aerogel materials. For all studied CAG samples, there are two broad peaks observed at about 24° and 44° 2θ, which correspond to the (002) and (101) crystal planes in the carbon structure. This indicates that the synthesized carbonaceous materials are amorphous with some contributions of graphene-like domains [31]. We also observed the interplanar spacing d_002_ growth, reaching values of 0.3949, 0.3959, 0.4016, 0.4023, nm for CAG_ potato starch (PS)+1%GA, CAG_PS+5%GA, CAG_PS+10%GA, CAG_PS+20%GA materials, respectively (Figure 1b). The same tendency, but less significant, was observed for the d_101_ values. The results suggest the deterioration of CAG graphitization and indicate the presence of more disordered graphitic domains as the GA amount rises in the sample. However, as can be noticed, this effect is not observed when we compare CAG_PS+1%GA and pristine CAG_PS samples.

The CHN analysis shows no significant effect of the gum arabic addition on the elemental composition of the studied samples (Table 1). With the growth of GA in the sample, the carbon content slightly decreases as follows: 91.8%, 91.0%, 90.8%, 90.7%, 89.1% for the CAG_PS, CAG_PS+1%GA, CAG_PS+5%GA, CAG_PS+10%GA, CAG_PS+20%GA materials, respectively. The amount of nitrogen remains almost unchanged between the modified samples and is equal to about 0.1%.

**Figure 2:**
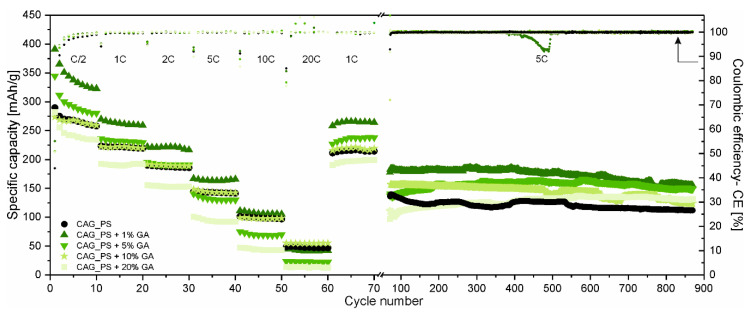
Galvanostatic charge–discharge tests under the different current loads with the additional long-term cycling for GA-structured carbon aerogels.

The rating (at different C-rates ranging from C/2 to 20C) and long-term cycling performance (at 5C-rate) of the obtained carbonaceous anode materials based on the potato starch precursor modified with different amounts of gum arabic (CAG_PS+GA samples) are displayed in Figure 2. The results of carbon aerogel derived only from potato starch (CAG_PS sample) are also included as the reference.

There is no doubt that gum arabic influences the overall electrochemical characteristics of CAG_PS and the comparison of the materials at different C-rates clearly indicates that the direction of changes is dependent on the quantity of the additive. As a matter of fact, for a small amount of GA (1% and 5%), the CAG_PS exhibits enhanced cycling performance. However, it needs to be added that in the case of a CAG_PS+1%GA electrode, the improvement concerns almost the whole range of applied C-rates (except 10C and 20C-rates, when the capacities are comparable to these of the reference electrode, amounting to approximately 100 and 50 mAh/g, respectively), while for the CAG_PS+5%GA, the higher capacities are observed only for the lower C-rates (C/2 and 1C). The opposite situation takes place for CAG_PS+20%GA which demonstrates a deteriorated performance. As regards the CAG_PS+10%GA, the electrochemical tests do not reveal any significant changes in reference to the CAG_PS behavior, since both cells show similar working parameters in terms of the capacity and reversibility of electrochemical processes. The charge capacities for the 1st cycle (under C/2 current rate) are as follows: 290, 391, 344, 274 and 282 mAh/g for the CAG_PS, CAG_PS+1%GA, CAG_PS+5%GA, CAG_PS+10%GA and CAG_PS+20%GA, respectively. In addition to the fact that CAG_PS+1%GA discloses the highest values of specific capacity from the entire group of tested electrode materials, it is also characterized by the highest coulombic efficiency (55%) in the initial cycle that can be translated into the lowest capacity loss ascribed to the electrolyte decomposition and the subsequent formation of the solid electrolyte interface (SEI) layer on the electrode surface. For the remaining studied cells, the coulombic efficiency for the 1st cycle is slightly lower and reaches 53% for CAG_PS+10%GA, 51% for CAG_PS+5%GA and CAG_PS+20%GA and 44% for CAG_PS. Although the irreversible Li^+^ consumption in the 1st cycle is quite large, following the initial discharge–charge process, the coulombic efficiency stabilizes after several cycles and rises to almost 100% for all cells. Some deviations from this value can be observed for the initial cycles of the subsequent series of electrochemical cycling, especially under higher current rates. This may be caused due to the fact that stronger polarization (resulting from increasing current densities each time) makes the new regions in material structure available for lithium ions (that are normally inaccessible). Such an irreversible incorporation of lithium ions into the materials structure will reduce the coulombic efficiency. There is also need to mention that this effect may also result from the partial decomposition of the passivation layer, which can occur especially when the higher current rates are applied.

Another point of view in the discussion is provided by the outcomes of long-term discharge–charge tests (Figure 2). They not only confirm that CAG_PS+1%GA is characterized by the greatest electrochemical properties, but also indicate that the addition of gum arabic has in general a beneficial effect on CAG_PS. It needs to be noticed that although CAG_PS material presents similar specific capacities in comparison to CAG_PS+5% GA and CAG_PS+10% GA under 5C current rate in rate performance tests, during long-term cycling all the modified materials (CAG_PS+GA samples) outperform CAG_PS, being more stable and disclosing higher capacities. It follows that the conditions in which all materials were tested next (10C and 20C) may be too harsh for some of investigated samples. Apparently, an addition of GA successfully prevents the cycling performance deterioration of CAG materials, after applying such high current rates. It is observed that the long-term cycling of CAG_PS shows a swift material capacity deterioration, from the beginning, while CAG anodes with GA additive exhibit an improved stability and seem to be undamaged after extreme current load application. The addition of GA provides a possibility to functionalize starch-based CAG materials for either long-term cycling or high-performance versatility. The charge capacities delivered by the cells containing CAG_PS, CAG_PS+1%GA, CAG_PS+5%GA, CAG_PS+10%GA and CAG_PS+20%GA after 870 cycles at 5C-rate are maintained at 112, 155, 149, 124 and 131 mAh/g in the given order. As regards the capacity retention after 870 cycles, the best results (in relation to the 31st cycle charge capacity) are noticed for CAG_PS+20%GA and CAG_PS+5%GA samples (over 100% due to the increase in the capacity during conducted experiments). For CAG_PS+1%GA and CAG_PS+10%GA, 93% and 86% of charge capacity from the 31st cycle is retained at the end of tests, implying the excellent efficiency and cycling stability of the modified electrodes. In contrast, the capacity retention value for CAG_PS material is equal to 76%.

**Figure 3:**
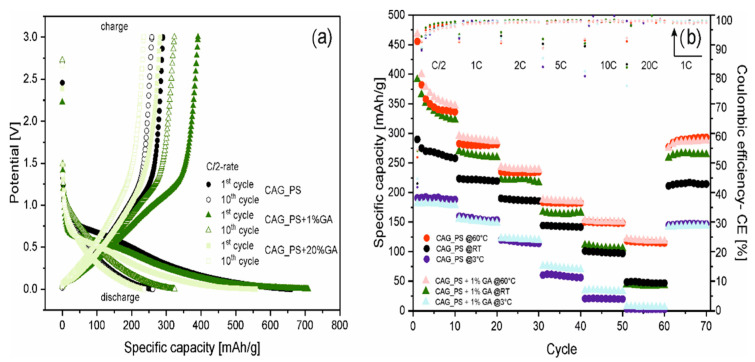
(**a**) Charge–discharge voltage curves for the first and tenth cycles of CAG_PS, CAG_PS+1%GA and CAG_PS+20%GA @RT and @C/2-rate; and (**b**) the galvanostatic charge–discharge tests at various temperatures for the CAG_PS+1%GA and CAG_PS as a reference.

Figure 3a illustrates the potential curves for galvanostatic charging and discharging processes at C/2 current rate for the 1st and 10th cycles of three selected samples (CAG_PS, CAG_PS+1%GA and CAG_PS+20%GA). The presented voltage profiles of the prepared carbon aerogels (for both the base and modified materials) reveal the common characteristics of disordered carbons containing graphite domains with aforementioned highly irreversible capacity, as well as noticeable charge and discharge voltage hysteresis and indistinguishable plateaus. Furthermore, regardless of the introduction of gum arabic to the CAG_PS system, the nature of the potential curves remains unchanged. Nevertheless, the impact of the additive on the electrochemical operation of starch-based carbon aerogels is unarguable. It can be supposed that the modification of starch precursor with GA favorably affects its physical and rheological properties, causing the stabilization of organic (starch) aerogel structure. This in turn contributes to the complete improvement of carbon aerogels’ functional properties and makes the features of the end product more preferable.

In addition to the standard rating and long-term cycling performance, the most distinctive CAG_PS+1%GA as well as the reference CAG_PS samples were further analyzed by performing the charge/discharge tests using different current rates (the applied multi-step procedure is analogous to that previously used) at various temperatures (3 °C, 20 °C and 60 °C). The results of the conducted investigation are depicted in Figure 3b. First of all, it should be pointed out that the highest values of specific capacities (456 and 468 mAh/g for CAG_PS and CAG_PS+1%GA at 1st cycle under C/2 current rate) are noted for the cells measured at raised temperatures, which is connected with the improved kinetics of active materials. A much more pronounced influence of higher temperature is visible for the CAG_PS material and for both tested electrodes in case of higher C-rates (10C, 20C-rates). In fact, considering the current rates, a significant increase in capacity is perceived at elevated temperatures (from about 100 to 150 mAh/g under 10C-rate and from 50 to 120 mAh/g under 20C-rate for both electrodes). The capacity retention of the cells (CAG_PS and CAG_PS+1%GA, respectively) at 60 °C after 70 cycles equals to over 100% and 97% of their 11th charge capacity. A dissimilar effect occurs for the cells measured at 3 °C, for which the decrease in the capacity in relation to the cells tested at room temperature is remarked. Furthermore, no significant changes are noticeable between CAG_PS and CAG_PS+1%GA materials.

**Figure 4:**
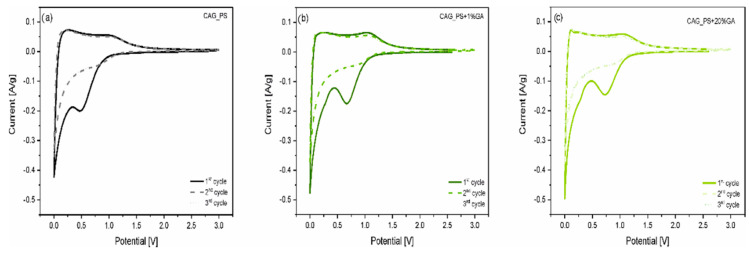
Cyclic voltammetry (CV) curves of (**a**) CAG_PS (**b**) CAG_PS+1%GA and (**c**) CAG_PS+20%GA-based half cells at a scan rate of 0.1 mV/s in the voltage range of 0.001–3 V.

In order to investigate the electrochemical processes running inside the Li-ion cell, the cyclic voltammetry measurements for Li/Li^+^/CAG_PS, Li/Li^+^/CAG_PS+1%GA and Li/Li^+^/CAG_PS+20%GA were carried out in the voltage range from 0.001 to 3.0 V at 0.1 mV/s scan rate (Figure 4). As can be observed, for all the studied cells, there is a sharp reduction peak around 0 V which is associated with the reversible insertion of lithium into carbon structures. The oxidation peak around 0.2–0.25 V corresponds well to the lithium extraction from carbons. However, during the first discharge process, a broad irreversible peak appears for the investigated samples, at different potentials from around 0.6 to 0.8 V. It can be assigned to the SEI layer formation on the carbonaceous surface and is responsible for the large irreversible capacity of the initial cycle. It is also noticed that the other two CV curves (for 2nd and 3rd cycles) overlapped, which indicates the good cycling stability of the analyzed materials. Furthermore, there are no additional peaks showing the electrochemical activity of the gum arabic itself, which confirms its role as a structuring additive. All these results are consistent with the galvanostatic charge/discharge outcomes.

## 4. Conclusions

As it is known, gum arabic is commonly used in the food industry as an emulsifying agent, which structures and thickens the starch-based systems by changing their parameters. It seems that when it is applied to Li-ion cells, GA also plays such a role. Gum arabic affects starch precursor rheological properties (as the amount of GA increases, the gelatinization process goes faster) causing the stabilization of organic aerogel structure and probably allows to achieve the desirable structural, textural and morphological characteristics of modified carbon aerogel samples for energy storage. The presented results suggest that GA may act as a structural promoter improving precursor rearranging for the graphitic domains’ nuclei growth. If the GA addition is above 1% *w*/*w*, the domains disorder becomes slightly larger, due to higher amount of concurrent nuclei growth, what causes a gradual deterioration in modified aerogels’ electrochemical properties with an increase in a gum amount. However, for CAG_PS+1%GA, there are no visible changes in the organization of the graphitic domains in comparison to pristine CAG_PS. It is possible that the appropriate ordering in the structure, the effect of which is not visible on XRD, most probably low range local ordering, has been achieved. What is more, for this sample, the operational parameters such as specific capacity and stability are significantly improved. There is also a need to mention that GA does not show the electrochemical activity itself and seems not to be involved directly in the electrochemical work. Therefore, all the observed differences in the electrochemical characteristics of the samples do not result directly from the processes taking place in the cell, but from the specific structure, morphology and porosity of the used, modified carbon materials, which of course influences the overall cell performance. However, some questions still remain unclear and it needs to be resolved during further, thorough research.

## Figures and Tables

**Table 1:** Elemental analysis results of pristine (CAG_PS) and modified (CAG_PS + GA) samples.

Sample Name	N (wt.%)	C (wt.%)	H (wt.%)
CAG_PS	0.4	91.8	1.7
CAG_PS + 1% GA	0.1	91.0	1.5
CAG_PS + 5% GA	0.1	90.8	1.5
CAG_PS + 10% GA	0.1	90.7	1.3
CAG_PS + 20% GA	0.1	89.1	1.4
